# Left atrial functional recovery precedes volumetric reverse remodeling after TAVI by speckle tracking echocardiography

**DOI:** 10.1038/s41598-026-42221-8

**Published:** 2026-04-07

**Authors:** Guishen Li, Nannan Liu, Wei Sun, Kai Xu, Bin Wang, Weiwei Zhou, Yujia Han

**Affiliations:** Institute of Cardiovascular Diseases of PLA, General Hospital of Northern Theater Command, Shenyang, 110016 China

**Keywords:** Speckle-tracking echocardiography, Transcatheter aortic valve replacement, Aortic stenosis, Left atrium, Strain, Reverse remodeling, Cardiology, Diseases, Medical research

## Abstract

The temporal sequence of left atrial (LA) functional and structural remodeling after transcatheter aortic valve implantation (TAVI) for severe aortic stenosis (AS) remains inadequately characterized. We aimed to evaluate the mid-term changes in LA mechanics and volume using speckle-tracking echocardiography (STE). In this prospective observational study, 162 patients with severe AS undergoing TAVI were enrolled. Echocardiography was performed at baseline, 1, 6, and 12 months post-procedure. LA reservoir strain (LASr), conduit strain (LAScd), and pump strain (LASct) were measured by STE. LA volumes and the LA volume index (LAVI) were also assessed. LA strain parameters (LASr, LAScd, LASct) improved significantly at 1 month post-TAVI (all *P* < 0.05) and continued to improve through 12 months. LAVI also decreased significantly at 1 month (34.3 ± 6.6 ml/m² to 28.5 ± 5.9 ml/m², *P* < 0.05). However, the absolute LA volumes (LAVmax and LAVmin) showed significant reduction only at 6 months post-TAVI (*P* < 0.05). After TAVI, LA functional improvement, assessed by STE, occurs early and with greater magnitude than volumetric changes. While LAVI decreases promptly—likely reflecting early hemodynamic unloading—reductions in absolute LA volumes are delayed. STE-derived LA strain serves as a sensitive early marker of LA adaptation following relief of outflow obstruction.

## Introduction

Transcatheter aortic valve implantation (TAVI) has become a standard treatment for patients with severe aortic stenosis (AS) who are at high surgical risk^1^. While the benefits of TAVI on left ventricular (LV) function and symptoms are well-established^2^, the subsequent adaptation of the left atrium (LA)—a key chamber modulating LV filling and a potent prognostic marker in cardiac disease^3^—is less well defined.

Conventional echocardiographic assessment of the LA relies predominantly on volumetric indices, which reflect chronic pressure overload but may be slow to change after intervention. Speckle-tracking echocardiography (STE) enables a more sensitive, quantitative evaluation of myocardial deformation^4^. LA strain parameters derived from STE have been shown to correlate with LV filling pressures and outcomes in heart failure and valvular heart disease^5,6^. However, the temporal pattern of LA functional recovery relative to structural reverse remodeling after TAVI has not been prospectively characterized in a mid-term setting.

The objective of this study was to serially evaluate LA mechanics and volume using STE at multiple time points up to 1 year following TAVI, to test the hypothesis that improvement in LA strain precedes the reduction in LA volume.

## Methods

### Study design and participants

This prospective observational study was conducted at the General Hospital of Northern Theater Command. From February 2023 to June 2024, consecutive patients with severe symptomatic aortic stenosis (AS) scheduled for transcatheter aortic valve implantation (TAVI) were screened. The diagnosis of severe AS was based on current guidelines^7^. Exclusion criteria included more than moderate other valvular heart disease, persistent atrial fibrillation, hypertrophic cardiomyopathy, inadequate image quality for speckle-tracking echocardiography (STE) analysis, and life expectancy < 1 year.

This study was conducted in accordance with the Declaration of Helsinki and relevant national and international guidelines and regulations. The Institutional Review Board of the General Hospital of Northern Theater Command approved the study protocol, and written informed consent was obtained from all participants or their legal guardians.

### Echocardiographic examination and analysis

Comprehensive transthoracic echocardiography was performed using a Philips EPIQ 7 C ultrasound system (X5-1 transducer) at four time points: pre-TAVI (baseline), and 1, 6, and 12 months post-TAVI.

Standard 2D images from the apical 4-chamber, 2-chamber, and long-axis views were acquired. LA volumes were measured using the biplane area-length method to obtain LA maximum volume (LAVmax), LA minimum volume (LAVmin), and LA volume index (LAVI).

**Fig. 1:**
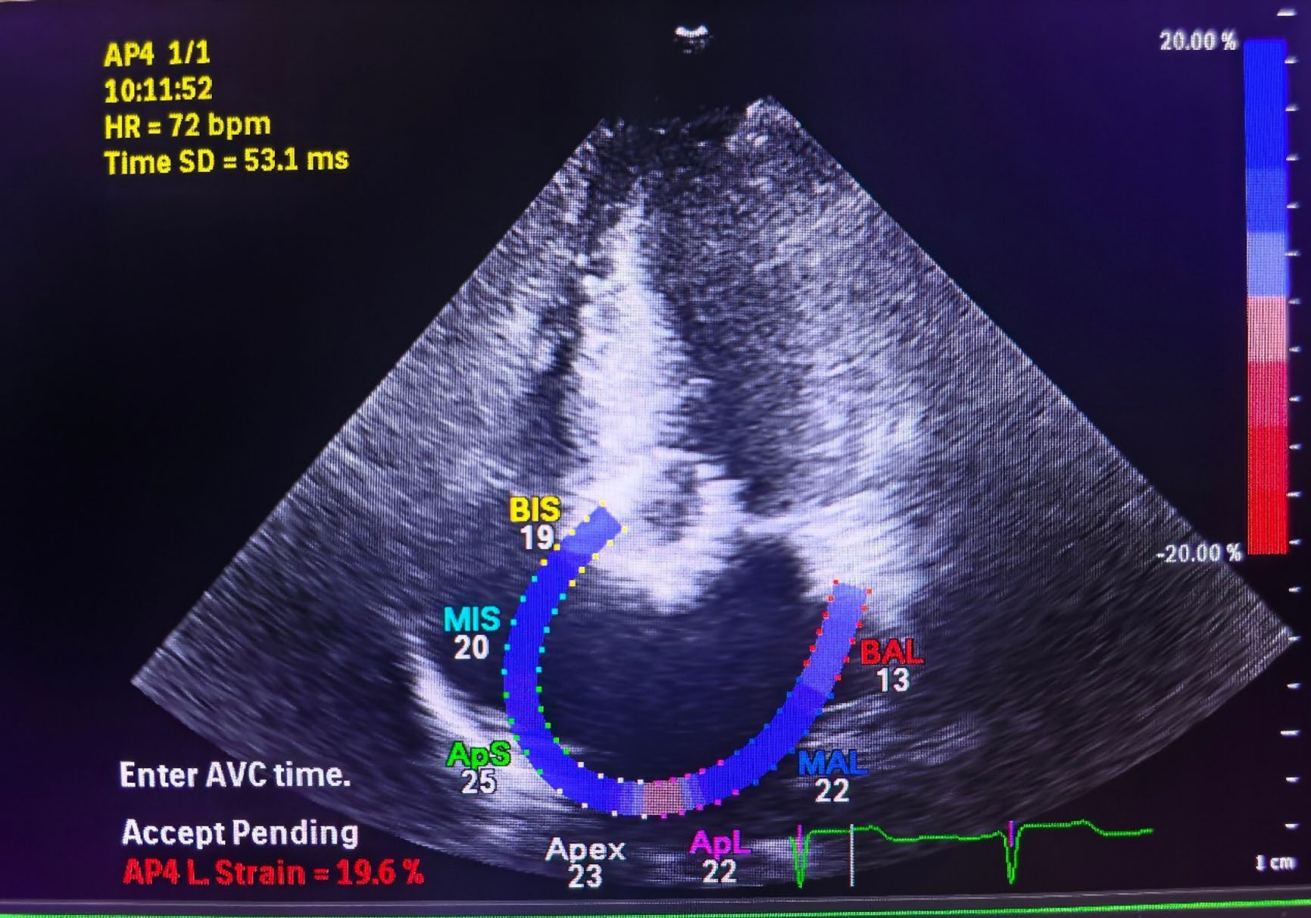
Speckle-tracking echocardiography for left atrial strain analysis.

For STE analysis, dedicated software (QLab version 10.8, Philips) was used. The LA endocardium was manually traced in the apical 4-chamber view at end-systole. The software then automatically tracked speckle patterns to generate LA strain curves. Global LASr, LAScd, and LASct were obtained from the average values of all segments (Fig. 1). All analyses were performed by two experienced sonographers blinded to the clinical data and time point. The average of three cardiac cycles was used.

### Statistical analysis

Statistical analyses were performed using SPSS version 25.0. Continuous variables are expressed as mean ± standard deviation. Comparisons of parameters across multiple time points were performed using repeated-measures analysis of variance (ANOVA). A *P* value of < 0.05 was considered statistically significant.

## Results

### Baseline characteristics

A total of 162 patients (98 men, 64 women; mean age 75.2 ± 6.5 years) were included. Baseline clinical characteristics are summarized in Table 1. All patients had severe AS, and the majority were in NYHA functional class III or IV.

**Table 1:** Baseline characteristics of the study population (*n* = 162).

Characteristic demographics	Value
Age, years	77.2 ± 6.5
Male gender	98 (60.5%)
Body surface area, m²	1.72 ± 0.18
Comorbidities	
Hypertension	98 (60.5%)
Diabetes mellitus	42 (25.9%)
Coronary artery disease	32 (19.8%)
Chronic kidney disease	28 (17.3%)
Chronic obstructive pulmonary disease	25 (15.4%)
Clinical Parameters	
Systolic blood pressure, mmHg	168.5 ± 24.3
Diastolic blood pressure, mmHg	82.3 ± 12.6
Heart rate, beats/min	72.4 ± 11.8
NYHA functional class	
II	25 (15.4%)
III	102 (63.0%)
IV	35 (21.6%)
Biochemical Markers	
NT-proBNP, pg/mL	3961.0 ± 1775.8
Creatinine, mg/dL	1.25 ± 0.42
Hemoglobin, g/dL	12.3 ± 1.6
Echocardiographic Parameters	
Aortic valve area, cm²	0.72 ± 0.15
Mean aortic gradient, mmHg	48.3 ± 12.6
Peak aortic velocity, m/s	4.5 ± 0.6
Left ventricular ejection fraction, %	56.3 ± 8.2
Left ventricular mass index, g/m²	125.6 ± 28.4
Medications	
Beta-blockers	118 (72.8%)
ACE inhibitors/ARBs	105 (64.8%)
Diuretics	92 (56.8%)
Statins	86 (53.1%)

Data are presented as mean ± standard deviation for continuous variables and number (percentage) for categorical variables. ACE = angiotensin-converting enzyme; ARB = angiotensin receptor blocker; NYHA = New York Heart Association.

### Temporal changes in LA strain and volume

**Fig. 2:**
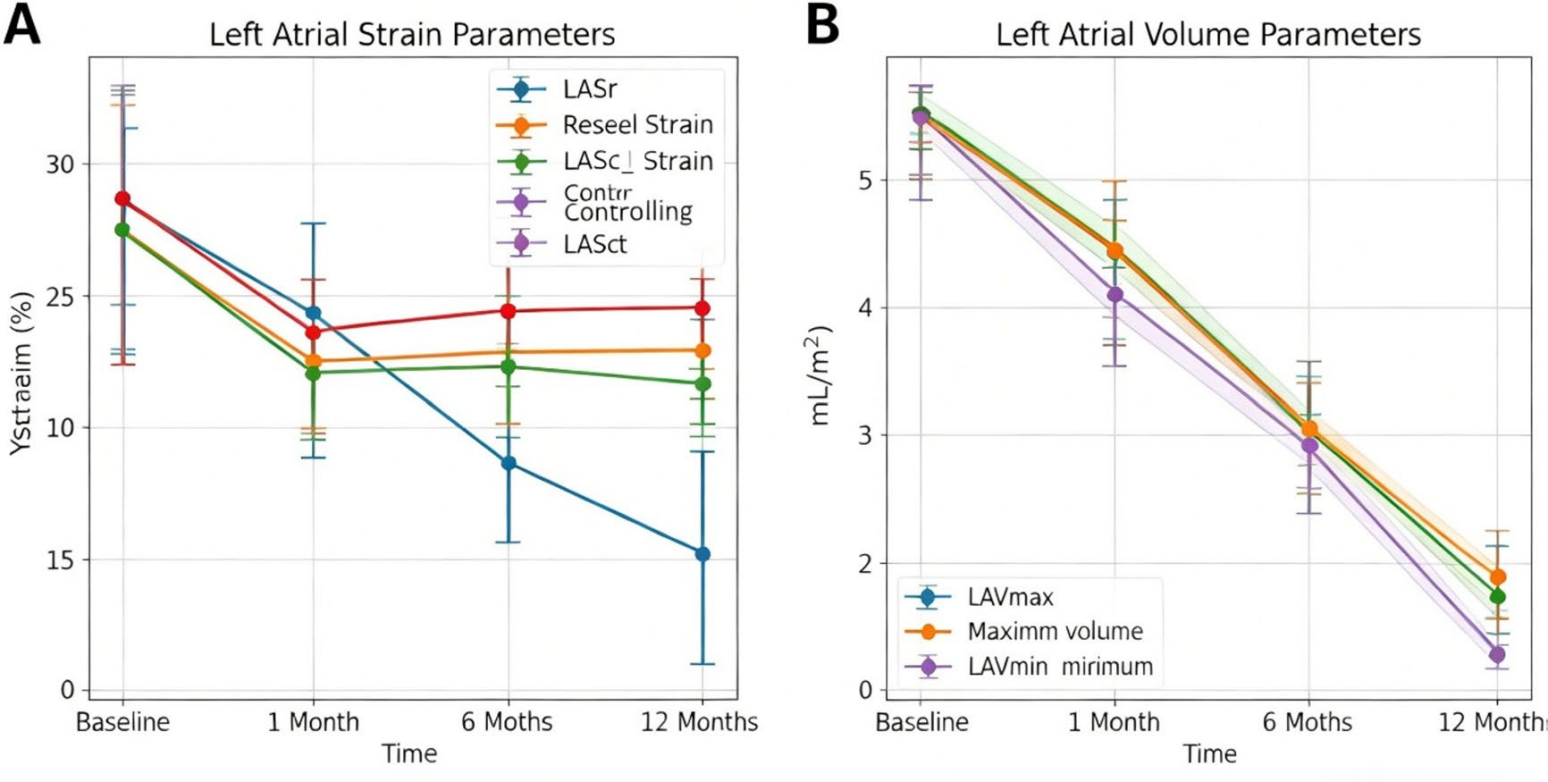
(**A**) Left atrial strain and (**B**) volume parameters at baseline, 1, 6, and 12 months post-TAVI.

The changes in LA strain and volume parameters over the follow-up period are detailed in Table 2. LA strain parameters (LASr, LAScd, LASct) improved significantly at 1 month post-TAVI (all *P* < 0.05) and continued to improve through 12 months (Fig. 2A). LAVI also decreased significantly at 1 month (34.3 ± 6.6 ml/m² to 28.5 ± 5.9 ml/m², *P* < 0.05). However, the absolute LA volumes (LAVmax and LAVmin) showed significant reduction only at 6 months post-TAVI (*P* < 0.05, Fig. 2B).

**Table 2:** Temporal changes in left atrial strain and volume parameters after TAVI.

Parameter	Pre-TAVI	1 Month Post-TAVI	6 Months Post-TAVI	12 Months Post-TAVI	F-value	*P*-value
LASr (%)	18.2 ± 4.6	24.9 ± 5.1*	28.5 ± 5.3*	31.2 ± 5.5*	68.925	0.023
LAScd (%)	10.5 ± 3.3	15.6 ± 3.9*	18.3 ± 4.1*	20.1 ± 4.3*	52.367	0.015
LASct (%)	8.4 ± 2.1	12.3 ± 2.6*	14.6 ± 2.8*	16.3 ± 3.0*	48.752	0.033
LAVmax (ml)	68.4 ± 11.5	65.6 ± 10.8	61.3 ± 9.8*	58.6 ± 9.2*	12.583	0.028
LAVmin (ml)	35.7 ± 8.2	33.2 ± 7.5	30.2 ± 6.7*	28.5 ± 6.3*	10.247	0.034
LAVI (ml/m²)	34.3 ± 6.6	28.5 ± 5.9*	25.1 ± 5.2*	23.4 ± 4.8*	45.692	0.041

Data are presented as mean ± standard deviation. *P < 0.05 compared with pre-TAVI.

## Discussion

This prospective study provides comprehensive evidence that LA functional recovery, quantified by STE-derived strain parameters, occurs early and precedes volumetric reverse remodeling during mid-term follow-up after TAVI. The identified “function-first” pattern offers novel insights into the sequence of cardiac adaptation following relief of outflow obstruction in severe AS.

### Mechanisms of functional precedence

The rapid improvement in LA strain parameters within one month post-TAVI reflects immediate and profound hemodynamic and cellular-level changes. The primary mechanism involves **sudden afterload reduction**. Severe AS causes elevated LV systolic pressures, transmitted through increased LV diastolic pressures to the LA, resulting in chronic LA pressure overload^9^. TAVI instantly relieves aortic valve obstruction, substantially reducing LV pressures and consequently decreasing LA afterload^10^. This afterload reduction directly enhances LA myocardial contractile-diastolic efficiency, particularly evident in LAScd, which showed the most substantial improvement (93.3%), indicating early LV diastolic functional improvement directly benefiting LA performance^11^.

Furthermore, early functional enhancement involves **myocardial stress relief and microenvironmental optimization**. Chronic pressure overload maintains elevated LA wall stress, potentially compressing the subendocardial vascular plexus and creating relative ischemia while activating pro-fibrotic signaling pathways^12^. TAVI-induced load reduction decreases wall stress, potentially improving myocardial microcirculatory perfusion and restoring normal deformation capacity to “stunned” cardiomyocytes^13^. At the molecular level, pressure relief may initiate beneficial extracellular matrix remodeling by modulating the balance between matrix metalloproteinases (MMPs) and their tissue inhibitors (TIMPs), providing the molecular foundation for functional improvement^14^.

### Biological basis of delayed structural reverse remodeling

The delayed significant reduction in LA volumes until six months post-TAVI aligns with fundamental biological remodeling principles. LA reverse remodeling depends not only on load conditions but also on **chronic extracellular matrix reorganization**^15^. Long-term pressure overload promotes myocardial fibroblast activation, leading to excessive collagen deposition and interstitial fibrosis, increasing LA stiffness^16^. Although TAVI removes the mechanical stress driving fibrogenesis, degradation of existing fibrotic tissue and collagen network remodeling require an extended period^17^. This process involves complex alterations in cellular signaling pathways, including downregulation of the TGF-β/Smad pathway and epigenetic regulation of fibrosis-related gene expression^18^.

The earlier significant reduction in LAVI compared to absolute volumes highlights its sensitivity in detecting initial structural changes, possibly because LAVI eliminates body size influences and more accurately reflects the LA’s true load status^19^. Additionally, early LAVI improvement may partly reflect immediate LV diastolic functional enhancement, whereas absolute volume changes require more comprehensive structural remodeling^20^.

### Integrating LA strain and volume findings

An interesting observation from our data is that both LASr and LAVI showed statistically significant changes as early as one month post-TAVI. This might initially appear to contradict our conclusion that functional improvement is more sensitive than volumetric remodeling. However, a closer examination reveals two important distinctions. First, the relative magnitude of change was substantially greater for LASr (Δ = +36.8%) than for LAVI (Δ = -16.9%) at one month. Second, and more importantly, the reductions in absolute LA volumes (LAVmax and LAVmin)—which represent true structural reverse remodeling—were not observed until six months post-procedure. The early reduction in LAVI is likely attributable to its indexed nature (correction for body surface area) and the immediate hemodynamic unloading following TAVI, rather than genuine structural regression of atrial myocardial mass. Therefore, while LAVI captures some early hemodynamic effects, STE-derived LA strain parameters provide a more direct and sensitive assessment of myocardial functional recovery, and absolute structural remodeling of the atrial chamber is indeed a delayed process.

### Clinical implications and translational value

Our findings carry significant clinical implications. First, they provide clinicians with a **sensitive early tool for assessing TAVI efficacy**. At one-month follow-up, when LA size may remain unchanged or LV ejection fraction (particularly in preserved EF patients) shows minimal alteration, significant STE-derived LA strain improvement serves as objective, quantitative evidence of procedural hemodynamic success^21^. This is especially relevant for heart failure with preserved ejection fraction patients, in whom traditional systolic function parameters often show limited change^22^.

Second, these parameters possess **prognostic potential**. Substantial evidence confirms that LA strain powerfully predicts atrial fibrillation, heart failure hospitalization, and all-cause mortality^23^. Notably, we observed LASr reaching approximately 31% at 12 months, approaching the lower limit of normal reference ranges. This suggests TAVI may not only alleviate symptoms but also genuinely modify the disease’s natural history. Based on this, we propose that the degree of LA strain improvement at six months post-TAVI could serve as a key time point for prognostic assessment^24^.

For clinical practice, our findings support integrating STE into **standardized post-TAVI follow-up protocols**. Specific recommendations include: during **early follow-up (1–3 months)**, focusing on strain parameter improvement to confirm initial efficacy; during **mid-term follow-up (6–12 months)**, comprehensively evaluating synergistic changes in strain and volumetric parameters; and during **long-term follow-up (beyond one year)**, establishing individualized functional baselines for monitoring late functional changes. This approach facilitates precision postoperative management and optimizes healthcare resource allocation^25^.

### Limitations and future directions

Several limitations warrant consideration. The single-center observational design may limit generalizability, requiring validation through multicenter studies. The absence of invasive hemodynamic data and specific fibrotic biomarkers constrains deeper mechanistic exploration. The lack of a conservative management control group prevents complete exclusion of other influencing factors. The sample size, while substantial, precluded subgroup analyses across different AS phenotypes. Extended follow-up is necessary to establish relationships between promising STE parameters and long-term clinical hard endpoints.

Future research should include establishing multicenter cohorts to validate the prognostic value of LA strain parameters, conducting comparative studies with cardiac magnetic resonance to determine STE’s accuracy in assessing myocardial fibrosis, exploring individualized postoperative management strategies guided by LA strain, and implementing extended follow-up studies to clarify relationships between functional improvement and long-term outcomes.

## Conclusions

This study demonstrates that after TAVI, LA functional improvement, assessed by speckle-tracking echocardiography, occurs early and with greater sensitivity than volumetric changes. LAVI decreases promptly, likely reflecting early hemodynamic unloading, whereas reductions in absolute LA volumes are delayed. STE-derived LA strain therefore serves as a sensitive early marker of LA adaptation following relief of outflow obstruction.

## Data Availability

The datasets generated and/or analyzed during the current study are not publicly available due to patient privacy and ethical restrictions but are available from the corresponding author (Nannan Liu) upon reasonable request.
